# Rare malignant ulcer related to primary intestinal diffuse large B-cell lymphoma: A case report

**DOI:** 10.1097/MD.0000000000018590

**Published:** 2020-02-07

**Authors:** Ning Jia, Yanping Tang, Yang Li

**Affiliations:** a Department of Gastroenterology, Tianjin Hospital of Integrated Traditional Chinese and Western Medicine; b Department of Diabetes, Tianjin Nankai District Hospital of Traditional Chinese Medicine, Tianjin, China.

**Keywords:** duodenal cancer, duodenal ulcer, endoscopy, primary intestinal diffuse large B-cell lymphoma

## Abstract

**Rationale:**

The specific pathogenesis of the diffuse large B-cell lymphoma(DLBCL)is still indefinite and argumentative. It is known that DLBCL is the most common type of non-Hodgkin's lymphomas (NHL). A lot of cases of DLBCL such as primary gastric diffuse large B-cell lymphoma(PG-DLBCL) are reported. However, primary intestinal diffuse large B-cell lymphoma(PI-DLBCL) is unusual.

**Patient concerns:**

We present a case of a 57-year-old male diagnosed in the Gastroenterology Department, which presented a bleeding duodenal ulcer with irregular borders.

**Diagnoses:**

The immunohistochemical staining showed: CD20(+++), CD10(+) and Ki-67>40%.

**Interventions:**

The patient was successfully treated by Poly-chemotherapy with R-CHOP (rituximab, cyclophosphamide, doxorubicin, vindesine and prednisolone).

**Outcomes:**

After 6 courses of chemotherapy treatment, the duodenal ulcer was completely healed by reviewing the UGIE.

**Lessons:**

Our report might give further strength to avoiding the erroneous and missed diagnosis for PI-DLBCL which is different from common duodenal ulcer.

## 1 Introduction

Diffuse large B-cell lymphoma is the most common type of Non-Hodgkin lymphomas (NHL) in adults which accounts for 30% to 40% of NHL.^[1]^ Diffuse large B-cell lymphoma is the commonest gastrointestinal lymphoma in China. Although the primary location of diffuse large B-cell lymphoma is different, it has been reported that the proportion of diffuse large B-cell lymphoma in gastrointestinal lymphoma is 50% to 60% in China. But primary intestinal diffuse large B-cell lymphoma is rare.^[2]^ Moreover, the incidence of primary intestinal diffuse large B-cell lymphoma (PI-DLBCL) is rarely reported. However, studies about PG-DLBCL, primary mediastinal DLBCL, primary central nervous system DLBCL, paraneoplastic erythroderma, and primary nasal DLBCL all are reported.^[3–6]^ The mechanism of action of DLBCL remains indefinite. The complications of DLBCL are also diverse and there is no unified standard. In this report, the patient was successfully treated by poly-chemotherapy with R-CHOP (rituximab, cyclophosphamide, doxorubicin, vindesine, and prednisolone).

## 2 Case report

**Figure 1:**
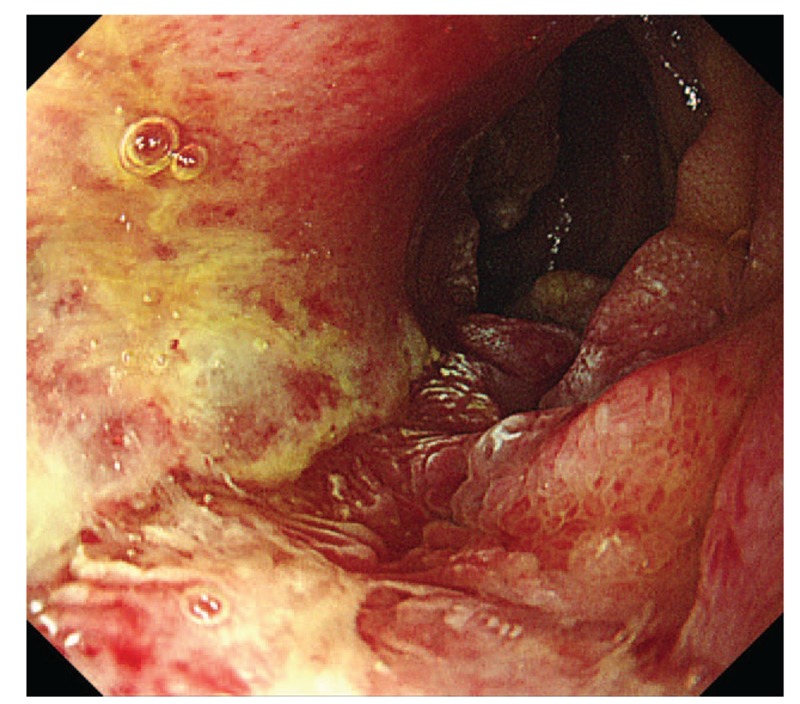
Upper gastrointestinal endoscopy revealing a 1.5 × 1.5 cm and big ulcer of descending part of duodenum with irregular border.

A 57-year-old man who presented with 20 days history of black color stool without treatment was taken to the hospital. The patient had upper abdominal pain, abdominal distension, and dizziness. But there were no other digestive syndromes containing acid reflux, heartburn, and vomiting. The patient had no previous medical history. Furthermore, there were no positive results by routine laboratory tests but exception of hemoglobin, 87 g/L and percentage of lymphocyte, 13.60%. In order to distinguish the nature of black stool, occult blood test is used because patients sometimes advocate taking bismuth drugs. And the stool occult blood test of this patient was positive. We took him for an upper gastrointestinal endoscopy (UGIE) examination that showed the following: a 1.5 × 1.5-cm and big ulcer of descending part of duodenum with irregular border. Congestion and edema existed around the mucosa of the duodenal ulcer. The surrounding mucosa is hypertrophied and devitalized, which differentiate it from peptic ulcer disease (Fig. 1).

**Figure 2:**
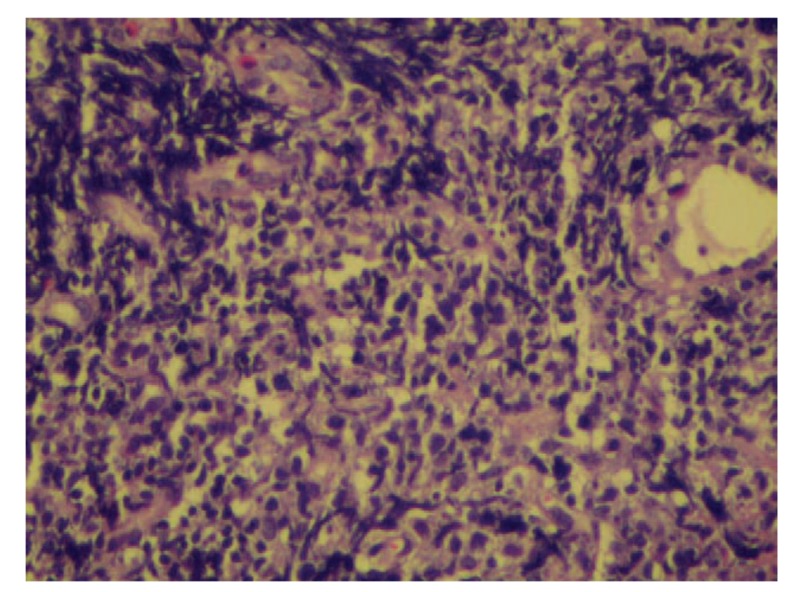
Histopathological observations of ulcer with diffuse proliferation and infiltration of small blue cells. (HE 4×).

First, the patient's symptoms of abdominal pain and distention were not significantly improved after a week with treatments consisting of esomeprazole for 80 mg daily (Intravenous Transfusion). Therefore, we took him for total abdomen Computed Tomography that showed a surprising result which was that the patient had a huge mass in the right mid-upper abdomen, which was considered to originate from the duodenum. So we considered this ulcer as a duodenal neoplasm. Afterwards, we were going to invite surgeons to consult patients to consider whether the patient should be treated with surgical treatment. However, the result of endoscopic biopsy came to our hands, which contained diffuse proliferation and infiltration of small blue cells which were found in the descending duodenum. Furthermore, immunohistochemical staining showed: CD20(+++), CD10(+), and Ki-67 >40%. However, we did tests including carcinoembryonic antigen, alpha fetoprotein, Carbohydrate antigen199, Cancer Antigen 724, ferritin, Cancer Antigen 242, Cancer Antigen 125, and prostate specific antigen, and they were all negative. And all observations showed that PI-DLBCL was identified (Fig. 2).

According to the treatment plan of Tianjin Institute of Hematology, the patient was treated with rituximab for 710 mg day 0, cyclophosphamide 1.4 g day 1, doxorubicin 60 mg day 1, vindesine 4 mg day 1, prednisolone 60 mg day 1–5. The patient's chemotherapy process went smoothly. No obvious adverse reactions were found. The patient showed great improvement in symptoms. After 6 courses of chemotherapy treatment, the duodenal ulcer was completely healed by reviewing the UGIE.

## 3 Discussion

Lymphoma is a malignant tumor originating from the lymphohematopoietic system. The most common types of lymphoma are NHL and Hodgkin lymphoma (HL). NHL lesions mainly occur in lymph nodes, spleens, thymus, and other lymphoid organs. They can also occur in lymphoid hematopoietic malignancies of lymphoid tissues.^[1,7,8]^ NHL's patients can also have symptoms including fever, emaciation, itchy skin, abdominal fullness and abdominal pain, indigestion, abdominal mass, gastrointestinal bleeding, and lymphadenopathy.^[1,9]^ DLBCL is the most common type of NHL. The stomach is a common site of DLBCL invasion.^[10]^ Although PG-DLBCL is reported,^[11]^ PI-DLBCL is unusual reported. And the basic pathogenesis of PI-DLBCL is not still clear, moreover there was no standard diagnostic criteria for PI-DLBCL. At present, the etiology of PI-DLBCL may include immune dysfunction, viral infection, and genetic factors.^[12]^

Nowadays, although it is sometimes difficult to distinguish between benign and malignant ulcers, our report is based on reliable pathological findings with definite diagnosis. Moreover, if we can perform chromoendoscopy or NBI-magnifying chromoendoscopy, we may be able to detect the malignant tumor early. Although we initially suspected duodenal cancer, we were able to correct the diagnosis in time. Therefore, it requires our clinicians to perform a large number of tissue-depth biopsies of ulcers which could be suspected of being tumors. According to the literature report, Cai et al^[13]^ report that combination of Rituximab and Lenalidomide is an effective therapy for the patients with PG-DLBCL. And our report also shows the effectiveness of poly-chemotherapy with R-CHOP which contributes to improvement in curing PI-DLBCL is very obvious.^[14]^

According to our report, we could put forward diagnostic criteria and therapeutic principles available for PI-DLBCL as follows: syndromes should include abdominal pain, abdominal distension, abdominal discomfort, or gastrointestinal bleeding; CD20(+) and Ki-67(+) should be detected from immunohistochemical staining; the treatment of proton pump inhibitor is ineffective; adenocarcinoma of duodenum should be excluded; R-CHOP chemotherapy is recommended as the first choice for treatment.

In conclusion, we need more case reports and a long-term follow-up to make a greater contribution for diagnosis and treatment of PI-DLBCL.

## Author contributions

**Conceptualization:** Ning Jia.

**Visualization:** Yanping Tang, Yang Li.

**Writing – original draft:** Ning Jia.

**Writing – review & editing:** Ning Jia, Yang Li.

Ning Jia orcid: 0000-0002-5394-9004.
